# Supplementary material for: Vibronic coherence evolution in multidimensional ultrafast photochemical processes
Source: Nat Commun. 2019 Dec 9;10:5621. doi: 10.1038/s41467-019-13503-9 (PMC6901526; doi:10.1038/s41467-019-13503-9)
Supplement: Supplementary file 1 — Supplementary Information [file 41467_2019_13503_MOESM1_ESM.pdf]

SUPPLEMENTARY INFORMATION

Vibronic Coherence Evolution in Multidimensional Ultrafast Photochemical Processes

Gaynor *et al.*

Supplementary Note 1. Sample Preparation, Linear Absorption Spectra, Experimental Methods

1.1 Sample Preparation

The N3 dye (*cis*-Bis(isothiocyanato)bis(2,2'-bipyridyl-4,4'-dicarboxylato)ruthenium(II)) was purchased from Sigma Aldrich (703206 Alrdich) and used without further purification. A 40 mM sample was prepared in aqueous 300 mM NaOH (pH~13) solvent to fully deprotonate N3 and form $N3^{4-}$. Samples for transient-IR (tIR) experiments probing the ω_3=2000-2200 cm^{-1} region were prepared using deionized H_2O as solvent, while the tIR and 2D EV experiments probing ω_3=1250-1620 cm^{-1} were prepared in D_2O. A sample

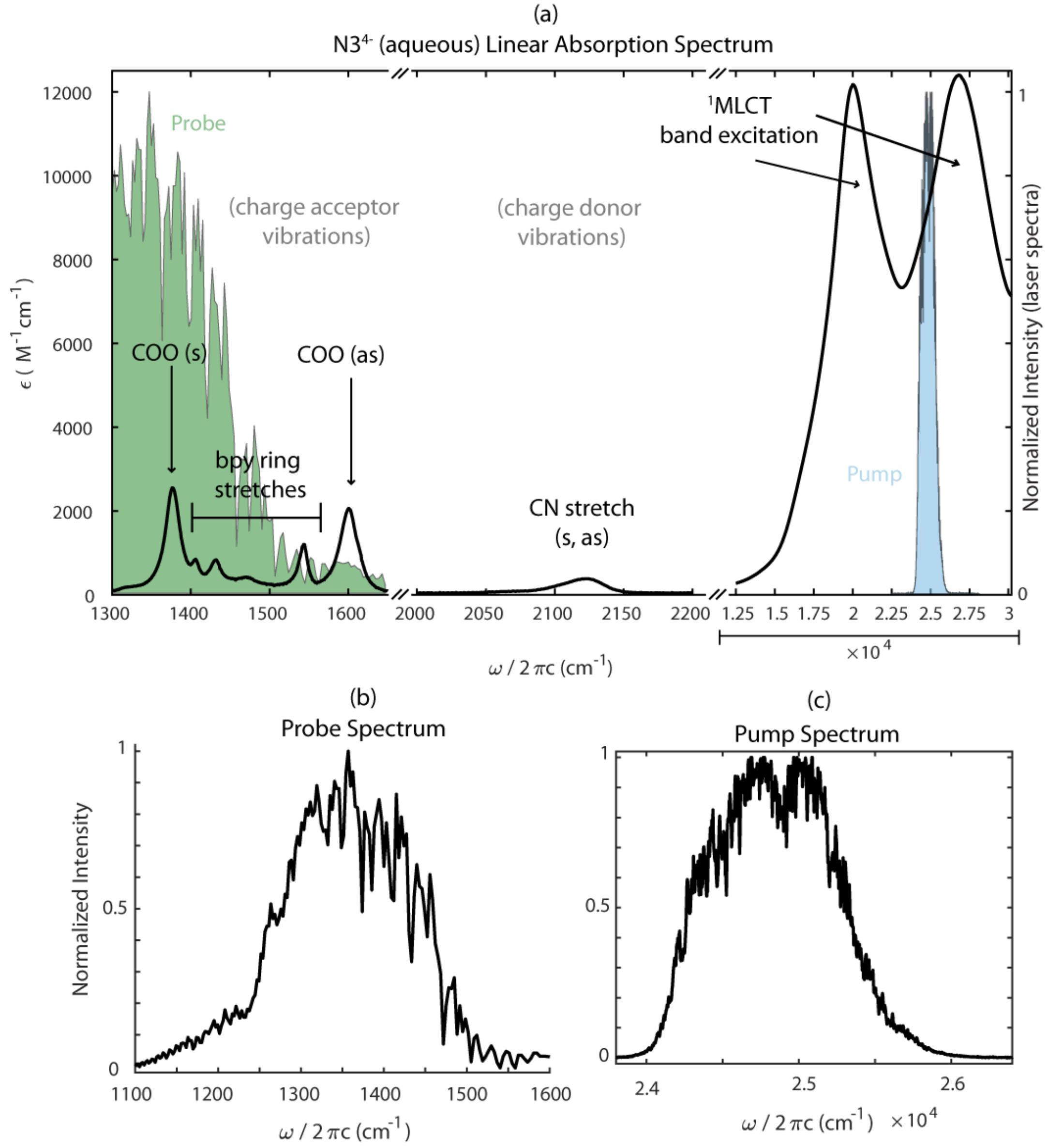

Supplementary Figure 1 Linear Absorption Spectrum of $N3^{4-}$ and Laser Pulse Spectra. (a) Aqueous $N3^{4-}$ linear absorption spectrum (combined solvent subtracted FTIR and UV-Vis spectra; laser pulse spectra overlaid). (b) Spectrum of the mid-IR probe pulse used in both tIR and 2D EV experiments. (c) Spectrum of the broadband UV pump pulse used in both tIR and 2D EV experiments.

flow cell (Harrick) with a 25 μm path length (25 μm Teflon spacer (Lebow) sandwiched between 1 mm and 2 mm CaF_2 windows) circulates sample solution driven by a peristaltic pump to refresh the volume of molecules illuminated by every laser shot (>1 kHz). Additionally, the sample cell was raster scanned in the plane orthogonal to the beam propagation to refresh the illuminated region of the sample cell window, which avoided sample build up inside the sample cell during long laser runs. The sample was enclosed in a septum-capped vial with needle connections to the flow cell tubing to minimize solvent evaporation during experiments; the samples with D_2O solvent were sealed carefully and stored in a desiccator. The $N3^{4-}$ purity was confirmed by UV-Vis and FTIR spectral measurements during several experiments using JASCO V-630 and JASCO FT/IR 4100 spectrometers with the same sample cell setup described above. UV-Vis and FTIR spectra taken before and after each experimental run confirmed there was no photodegradation during the tIR and 2D EV experiments. This sample configuration yields an electronic absorption optical density (OD) $\leq$ 1.0 OD across the frequency range spanned by our pump pulse. The vibrational absorption OD (without solvent subtraction) of the carboxylate stretches is $\sim$ 0.45 OD with D_2O solvent contributions of ~0.2 OD throughout the fingerprint region.

1.2 Linear Absorption Characterization (UV-Vis and FTIR)

The electronic absorption spectrum of the aqueous $N3^{4-}$ in Supplementary Figure 1(a) includes two singlet metal-to-ligand charge transfer (MLCT) excitations at 20000 cm^{-1} (500 nm) and 26880 cm^{-1} (372 nm). As shown in our earlier work on this compound,[1] many singlet MLCT states underlie these broad absorption bands forming a dense excited state manifold. The tIR and 2D EV experiments discussed in the main text excite the red edge of the higher energy ^{1}MLCT absorption band.

The solvent subtracted vibrational absorption spectrum of the aqueous $N3^{4-}$ in Supplementary Figure 1(a) show the ground electronic state vibrational features for both the Ru-$(NCS)_2$ charge donor region and the dicarboxybipyridine (dcbpy) ligand charge acceptor region. The CN symmetric and asymmetric stretches of Ru-$(NCS)_2$ are overlapped in the vibrational peak appearing at 2116 cm^{-1}. The carboxylate asymmetric stretch (1596 cm^{-1}) and symmetric stretch (1375 cm^{-1}) feature prominently for the charge accepting dcbpy ligand. A number of bipyridine (bpy) localized ring modes are also present in the 1400-1550 cm^{-1} region.[1]

1.3 Transient-IR and 2D EV Instrumental Setup

The general instrumental configuration has been described elsewhere in great detail.[1,2] All pulses used in the experiment are derived from the fundamental output of a Ti:Sapphire regenerative amplifier (Spectra Physics Spitfire XP Pro; 800 nm, 4.0 W, ~40 femtoseconds (fs), 1 kHz). The broadband UV (BBUV) pump is generated by the second harmonic of a spectrally broadened portion of the 800 nm fundamental beam using a 100 μm BBO (Type I) crystal (Newlight Photonics). We use a similar multi-plate spectral broadening method reported by He *et al.*[3] and Lu *et al.*[4] The spectral broadening of the fundamental beam is achieved by placing three thin BK7 windows (140 μm thick) just after the beam waist of a focusing 250 μJ/pulse portion of the 800 nm fundamental beam in a transmissive Keplerian telescope geometry [focusing lens (BK7, AR 800 nm): f = 1 m, recollimating lens: (BK7 AR 600-1000 nm) f = 0.4 m]. The windows are oriented at approximately Brewster's angle to maximize beam transmission. At each transmission, self-phase modulation induces spectral broadening; by adjusting the inter-window spacing, the spectral broadening and pulse energy is optimized. The plates are mounted to allow for vertical translation to periodically refresh the incident spot being used for broadening which avoids accumulated photodamage to the windows and maintains a consistent BBUV spectral profile and pulse energy for the duration of experiments. After the spectrally broadened fundamental is frequency-doubled in the BBO crystal, a dichroic high reflective mirror isolates the BBUV pulse and routes it through a λ/2 waveplate and polarizer before entering a UV prism compressor (Newport 10SB10 prism pair). After the prism compressor, an

additional Keplerian telescope (UV fused silica plano-convex lenses, UV AR coated, f=100mm) is used to optimize beam collimation for further propagation.

The BBUV pump pulse is then amplitude and phase shaped using an acousto-optic programmable dispersive filter (UV-Dazzler, Fastlite) in both the tIR and 2D EV experiments. In these experiments, ~10 µJ / pulse of BBUV enters the Dazzler. To perform the 2D EV experiments, a collinear pump pulse pair is generated using the Dazzler and sequentially delayed over a time delay (τ_1) between the two pump pulses. The shaped UV pump pulse(s) exit the Dazzler with vertical (S) polarization and are routed to the sample area and focused with a UV-fused silica plano-convex lens (UV AR coated, f = 300 mm) through a machined hole in the first of two off-axis parabolic (OAP) mirrors. At the sample area, 240 nJ/pulse (S polarization, 220 µm 1/e^2 diameter) is used to excite the sample. The BBUV is chopped at 500 Hz to collect differential absorption (pumped – unpumped) spectra. The mid-IR is generated from difference frequency generation of the near-IR signal and idler outputs of an in-house built collinear two-stage optical parametric amplifier and routed through a delay stage (Newport ILS150PP) to control the τ_2 delay time. The mid-IR polarization incident to the sample is set at the magic angle (54.7^o) with respect to the BBUV pump polarization using a ZnSe holographic wire grid polarizer (ThorLabs WP25H-Z) to remove orientational contributions to the measured signal and collect purely the isotropic signal. The mid-IR probe pulse (480 nJ/pulse, 195 µm 1/e^2 diameter) is focused at the sample to be spatially overlapped with the pump beam. Another wire grid polarizer is set immediately after the sample cell, before the second of two off-axis parabolic mirrors, to be parallel to the incident mid-IR polarization which ensures measurement of only the isotropic signal components. The mid-IR signal is routed to a spectrometer (Jobin-Yvon Horiba Triax 190, 75 g/mm grating) and dispersed onto one stripe of a 2x64 mercury cadmium telluride pixel array (Infrared Systems). Signal within the instrument response due to pulse overlap at early

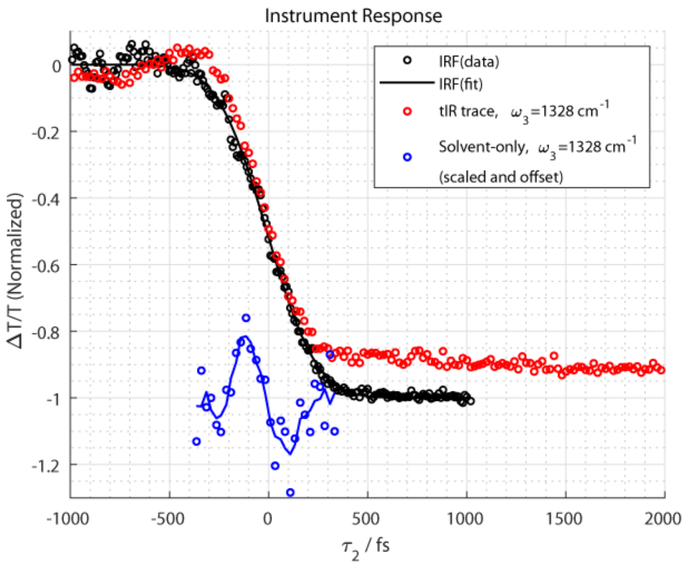

Supplementary Figure 2 The instrument response function is assessed using the non-resonant tIR signal of a 250 µm Si wafer (black). The N3^{4-} molecular response is observed outside of pulse overlap for τ_2 > 200 fs from the transient-IR trace for the ν_{COO} excited state absorption (ω_3=1328 cm^{-1}, red). The signals are normalized by the absolute value of the greatest magnitude signal and overlaid for comparison. The non-resonant solvent-only tIR signal (blue; circles are data and line is three-point moving average) is scaled and offset such that zero signal is ΔT/T = -1 for comparison; the solvent response diminishes for τ_2 > 200 fs, consistent with the decay of the Si signal and the onset of the N3^{4-} molecular signal.

times has diminished by $\tau_2 \cong$ 180-200 fs as estimated by the rise time of the non-resonant integrated pump-probe signal in a 250 μm Si wafer.

1.4 Data Acquisition

A well-averaged tIR data set used for 2D EV normalization is obtained by averaging 500 laser shots per difference spectrum while scanning over the range -1 ≤ τ_2 ≤ 200 picoseconds consecutively in the forward direction; 20 completed τ_2 scans are then averaged together for the final tIR data set. The entire tIR data set was collected in ~1.5 hours during the same laser run as the 2D EV data with the same experimental configuration to maintain consistency in as many systematic experimental parameters as possible. The tIR data set is divided by a well-averaged (5000 laser shots) sample spectrum collected with the BBUV pump blocked to obtain ΔT/T.

A single 2D EV spectrum at a given τ_2 delay time is collected by scanning τ_1 over the range [0:150] fs in 1.15 fs steps to expedite data acquisition using a partially rotated frame. The integrated field autocorrelation of the pump pulses scanned over τ_1 is collected and Fourier transformed to ensure that the shape of the excitation spectrum is not compromised due to under sampling. At each fixed τ_1 delay time and τ_2 delay time, 500 laser shots are averaged to obtain the differential absorption spectrum. The total range of pump-probe delay times collected in these 2D EV experiments are 10 ≤ τ_2 ≤ 2010 fs at 20 fs intervals. Six 2D EV scans were averaged for each τ_2 delay yielding a single averaged 2D EV data set. To minimize artifacts due to laser drift throughout the experiment, the entire τ_2 range studied was collected in two parts: first by scanning 250 ≤ τ_2 ≤ 2010 fs and then scanning 10 ≤ τ_2 ≤ 250 fs. Acquisition of the first range (250 ≤ τ_2 ≤ 2010 fs) was further divided into two separate experimental scans with 40 fs intervals: one for 250 ≤ τ_2 ≤ 2010 fs and the other for 270 ≤ τ_2 ≤ 1990 fs. Each of these subranges scanned with 40 fs intervals were scanned three times in the forward direction and three times in the reverse direction to further reduce artifacts from laser drift during the ~4.5 hour collection time of the 2D EV scan over the τ_2 range. Acquisition of the second range (10 ≤ τ_2 ≤ 250 fs) was collected in 20 fs intervals using the same approach, each scan taking ~1.5 hours of experimental collection time. Solvent-only 2D EV spectra were collected over 10 ≤ τ_2 ≤ 450 fs in 20 fs intervals to ensure no significant solvent features were present in the sample data (see Supplementary Figure 2(a) below). The pump power dependence of the 2D EV signal was measured to be linear with respect to pump power, ensuring that no multiphoton absorption signals were present in the tIR or 2D EV data (see Supplementary Figure 2(b)).

1.5 2D EV Data Processing – Fourier Transform over τ_1

Traditional FT data processing techniques are employed here.[2,5] A constant offset of the vibrational differential absorption signal due to the τ_1-independent pump-probe signal is initially subtracted. The τ_1-dependent differential absorption data is zero-padded to 2048 points, and a tanh apodization function is applied to smoothly transition the data to zero at long τ_1 times prior to the Fourier transform (FT).[6] The FT over the spectrally-detected, τ_1-dependent vibrational differential absorption data yields the electronic excitation spectrum, ω_1. The resulting 2D EV spectra are divided by a well-averaged (5000 laser shots) sample spectrum collected with the BBUV pump blocked to maintain the correct ω_3 spectral profile (proportional to ΔT/T, as in tIR). The well-averaged sample spectra used for division were collected throughout the experiment and indicate negligible change in spectral shape. Additional steps were also taken to correct for remaining instrumental noise to better isolate τ_2-dependent oscillatory signals, as detailed in the Supplementary Note 3.

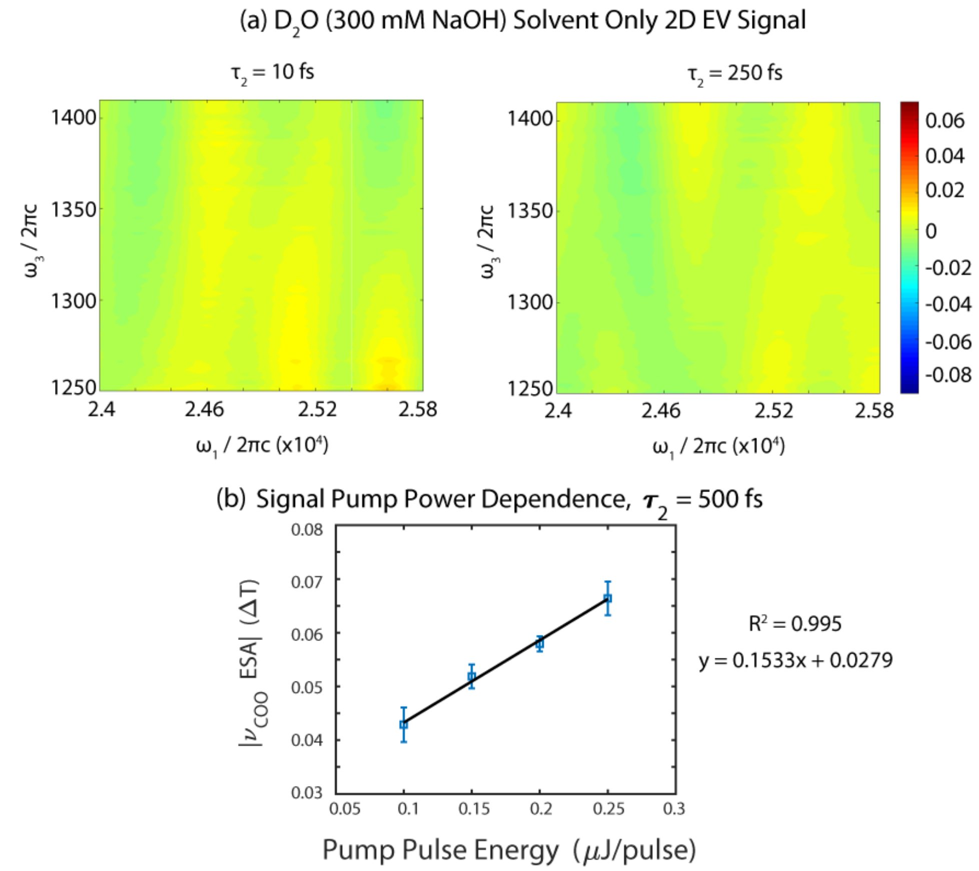

Supplementary Figure 3 Solvent Background and Signal Pump Power Dependence. (a) 2D EV spectra of the solvent (D_2O in 300 mM NaOH) collected in the same sample configuration as the $N3^{4-}$; no significant solvent contributions are present. (b) The magnitude of the ν_{COO} ESA (ω_3=1328 cm^{-1}) plotted at different pump pulse energies shows the data measured are linearly dependent on pump energy. Blue squares are average of five difference spectra with the error bars reflecting +/- 1 standard deviation from the mean; the black line is a linear fit with the fit parameters shown on the right.

Supplementary Note 2. Transient-IR Spectroscopy of $N3^{4-}$

We have previously identified the principal high-frequency vibrational signatures of the excited electronic states for both the charge donor and acceptor using parallel-polarized tIR spectroscopy of aqueous $N3^{4-}$.[1] Briefly, the charge donor CN stretches shift to lower frequency by 50-70 cm^{-1} as electron density transfers away from the Ru-$(NCS)_2$ and a stronger anharmonic coupling results in the observed ~20 cm^{-1} excited state splitting of the symmetric and asymmetric stretches. As electron density arrives at the acceptor, the bipyridine (bpy) ring vibrations shift to higher frequencies by ~45 cm^{-1} while the carboxylate (COO) stretches lower in vibrational frequency by ~50 cm^{-1} due to increased aromaticity of the dcbpy ligands. The excited state symmetric stretching carboxylate vibration (ω_3 = 1328 cm^{-1}), which we refer to as ν_{COO}, is a strong excited state vibration and rather isolated spectrally; it is the main high frequency vibration discussed for reasons below and in the main manuscript. Another spectrally isolated, electronically excited state vibration appears at 1271 cm^{-1} that is bpy-localized and referred to as ν_{BPY}. As noted in previous work,[1] the 1328 cm^{-1} carboxylate vibration is one of four excited triplet state symmetric stretching carboxylate normal modes in the 1300-1400 cm^{-1} frequency region; the other three are of higher

frequency and overlap with the four nearly-degenerate ground state carboxylate symmetric stretches centered at ω_3=1375 cm^{-1}.

The timescales of the excited state vibrational features measured by parallel polarized tIR (see Supplementary Figure 4 and Supplementary Table 1) show that the triplet state CN stretches are formed within the instrument response and consequently static for the duration of the experiment (200 picoseconds). The constant intensity and center frequency of the CN excited state vibrational features indicate negligible intramolecular structural reorganization of the charge donor for at least 200 picoseconds (ps) following photoexcitation and ultrafast intersystem crossing (ISC). This is consistent with other tIR studies on N3 which typically focused only on the CN stretching region, and with the fact that its ^{3}MLCT manifold has a lifetime on the order of nanoseconds. The ν_{COO} ESA also forms significant amplitude

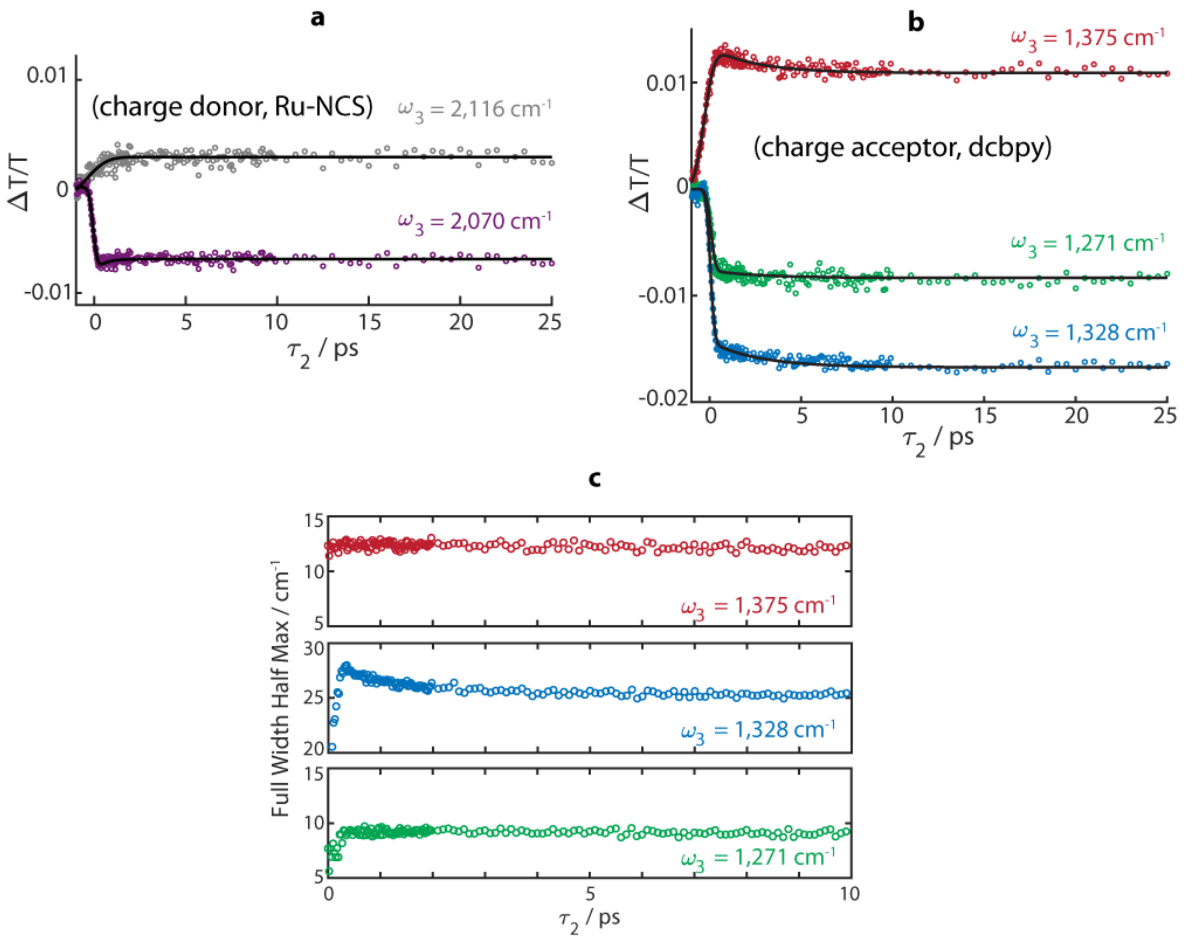

Supplementary Figure 4 Transient-IR Spectroscopy of N3^{4-}. (a) tIR time traces of the CN stretch region reports on the Ru-(NCS)$_2$ charge donor dynamics; ground state bleach (GSB) = 2116 cm^{-1} (gray circles), excited state absorption (ESA) = 2070 cm^{-1} (purple circles), time trace fits shown by solid black lines. (b) tIR time traces of the dcbpy ligand stretching region reports on the charge acceptor dynamics; carboxylate symmetric stretch GSB = 1375 cm^{-1} (red circles) and ESA = 1328 cm^{-1} (blue circles), bipyridine stretching mode ESA = 1271 cm^{-1} (green circles). (c) tIR line shapes identify which charge acceptor vibrations are sensitive to excited state relaxation, as shown by the τ_2-dependent full-width-at-half-max for all three peaks; the carboxylate symmetric stretch ESA is most sensitive to excited state charge transfer dynamics in the first few picoseconds of relaxation.

within the instrument response but then continues to grow on a 2.7 ± 0.8 ps timescale. Additionally, the carboxylate symmetric stretching GSB at 1375 cm^{-1} rises to its maximum intensity during the instrument response before decaying in amplitude on a 2.5 ± 0.8 ps timescale – effectively the same timescale as the ν_{COO} ESA growth. This is readily explained by the ESA dynamics of the overlapping carboxylate symmetric stretches noted above. Although much weaker, other bpy ESA features also grow in over the first several picoseconds of delay time.

While many charge acceptor vibrations display ESA signal growth during the triplet manifold relaxation, they are not all equally sensitive to the intramolecular structural dynamics involved with this relaxation process. The time-dependent tIR vibrational peak widths of the ν_{COO} and the ν_{BPY} features demonstrate that the ν_{COO} vibration is the most sensitive coordinate to triplet relaxation processes probed in our experiments as its full-width-at-half-maximum (FWHM) broadens to 28 cm^{-1} and narrows to 25 cm^{-1} within the first three ps of relaxation; whereas, the ν_{BPY} rises to 9 cm^{-1} within the instrument response and is unchanged thereafter. Together, the measured tIR timescales, line shapes, and negligible frequency shifts for both charge donor and acceptor ligand vibrations establish the photophysical picture of ultrafast triplet formation of $N3^{4-}$ and charge donation by the $Ru\text{-}(NCS)_2$ while the dcbpy ligand vibrations relax in a dense triplet manifold over the first several picoseconds post-excitation.

These tIR measurements provide an initial characterization of the charge donor-acceptor timescales and they identify excited state high-frequency vibrational coordinates involved with the ultrafast intramolecular charge transfer in $N3^{4-}$. However, these measurements necessarily convolve the intramolecular dynamics involving different electronic states which are resolved in the 2D EV experiment. We have already demonstrated using polarization-selective 2D EV spectroscopy that three different excited ^{1}MLCT electronic states are coupled with the CN charge donor vibrations, and that only two of these initially excited ^{1}MLCT states are coupled with the charge acceptor vibrational modes which likely provide avenues for ultrafast ISC and triplet relaxation in $N3^{4-}$.[1]

The time traces for the tIR data discussed in the manuscript and shown in Supplementary Figure 4 were fit to a Gaussian function convoluted with the sum of two exponentials to check that our data is consistent with reported N3 relaxation time scales, which has the form:

$$\begin{aligned} \mathrm{f}(t) = {} & A_1 \exp\left[\left(\frac{B}{4\sqrt{\ln(2)}\cdot\tau_{short}}\right)^2 - \frac{t-t_0}{\tau_{short}}\right] \times \frac{1}{2}\left(\operatorname{erf}\left[\frac{t-t_0}{B}\cdot 2\sqrt{\ln(2)} - \frac{B}{4\sqrt{\ln(2)}\cdot\tau_{short}}\right]+1\right)\cdots \\ & + A_2 \exp\left[\left(\frac{B}{4\sqrt{\ln(2)}\cdot\tau_{long}}\right)^2 - \frac{t-t_0}{\tau_{long}}\right] \times \frac{1}{2}\left(\operatorname{erf}\left[\frac{t-t_0}{B}\cdot 2\sqrt{\ln(2)} - \frac{B}{4\sqrt{\ln(2)}\cdot\tau_{long}}\right]+1\right). \end{aligned} \quad (1)$$

where A_1 and A_2 are amplitude factors for the two convolutions, B is the temporal FWHM of the Gaussian instrument response function, t_0 is time zero, τ_{short} is the ~picosecond time constant of one exponential decay and τ_{long} is the nanosecond time constant of the second exponential decay, erf is the error function. An additional constant offset (C) was also considered in fitting but did not significantly change the fitting results. Supplementary Table 1 gives the parameters obtained for the optimized fits of the time traces. The 95% confidence interval is given for each fitting parameter, all time values are in units of picoseconds.

Supplementary Table 1 *Transient-IR Fitting Parameters*

**ω_3 / cm^{-1}**	**A_{short} (ΔT/T)**	**τ_{short} /ps**	**A_{long} (ΔT/T)**	**τ_{long} /ps**	**B / ps**	**t_0 / ps**	**R^2 (adj)**
2116[b]	0.004	0.049	0.419	0.049	2.07	-0.435 ±0.761	0.6815
2070	-0.001 ±0.001	0.49 ±0.06	-0.0067 ±0.0002	1.6E4 ±1.0E5	0.44 ±0.06	-0.058 ±0.028	0.9723
2070[a]	0	0	-0.0069	16000	0.41	-0.087	0.9705
1375[b]	0.0026 ±0.0004	2.5 ±0.8	0.0109 ±0.00016	1.6E4 ±1.0E5	1.03 ±0.06	-0.284 ±0.021	0.9746
1328	0.0024 ±0.0003	2.7 ±0.8	-0.0168 ±0.0002	1.6E4 ±6.8E4	0.45 ±0.03	0.037 ±0.009	0.9936
1271	0.0007 ±0.0003	2.8 ±3.1	-0.0083 ±0.0003	16E4 ±1.6E5	0.47 ±0.07	0.070 ±0.022	0.9689

[a] *A_{short} and τ_{short} have been set to zero in a separate fit of the excited state CN stretch at 2070 cm^{-1} to show the lack of any significant decay component at early times, reflecting the instantaneous formation of the excited state charge donor.*

[b] *Ground state vibrational excitation at negative τ_2 times affect the accuracy of fits for early time components in ground state bleach features.*

Supplementary Note 3. τ_2 Fourier Transform Data Processing and Assessing Signal-to-Noise

3.1 Instrumental Noise Correction

The purpose of the FT analysis over the τ_2 delay time is to identify the frequencies of coherent oscillations that modulate the amplitude of the 2D EV peaks. These signatures reveal further information about the degrees of freedom coupling the vibronic states involved with the excited state charge transfer dynamics in $N3^{4-}$. Since these oscillations are very weak in our experiments, careful corrections for signal intensity fluctuations due to instrumental noise are required to isolate the coherent oscillations in 2D EV signal amplitude. As can be seen by comparing the blue and red time traces in Supplementary Figure 5 (bottom), instrumental noise can significantly overwhelm weaker oscillatory signals. We correct for this noise to isolate the τ_2 oscillations of interest by normalizing the raw 2D EV data for each τ_2 delay to a separate,

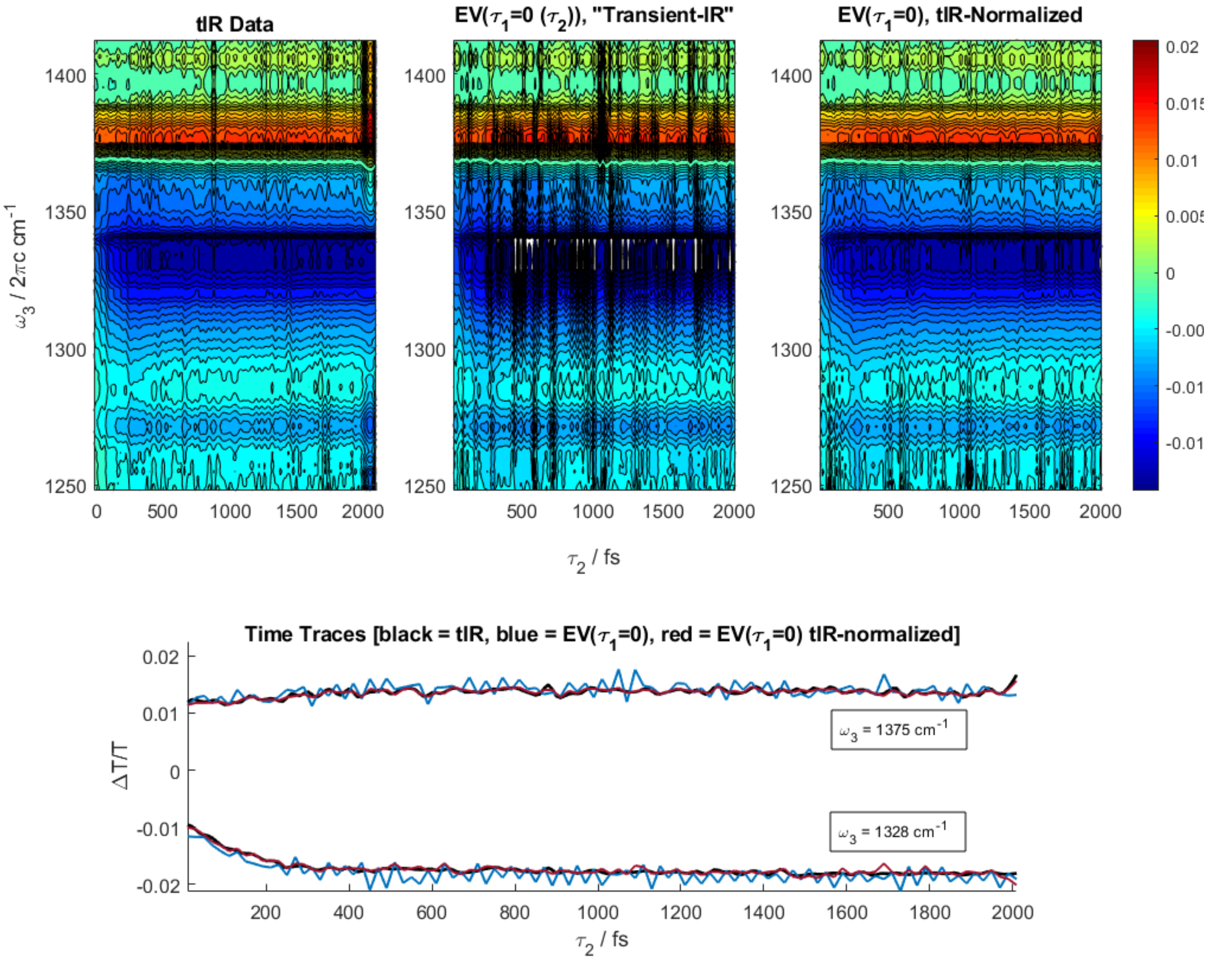

Supplementary Figure 5 Pre-FT Data Processing of τ_2-dependent 2D EV Data. (top, left) The cubic spline interpolated tIR spectrum collected independently, (top, center) the raw 2D EV spectra with τ_1 = 0 for all τ_2 delays, and (top, right) the 2D EV (τ_1 = 0) data corrected for instrumental noise by normalization to the tIR data set. (bottom) The time traces of the carboxylate GSB and ESA for the data sets show the improvement in signal quality after removing further instrumental noise due to fewer 2D EV scans averaged than in the tIR data set. The blue traces = 2D EV (τ_1=0) data set without normalization, red traces = with normalization, and black traces = interpolated tIR data (see discussion for details).

well-averaged tIR data set which was collected within the same laser run using an identical experimental configuration. The six 2D EV scans at a given τ_2 delay are first averaged in the time domain (τ_1). Then the differential absorption spectrum from the 2D EV data set for $\tau_1 = 0$ is normalized to the well-averaged tIR spectrum for the same τ_2 delay. Since these two data sets reflect an exactly identical experiment, the only difference between them is the experimental noise due to fewer scans averaged in the 2D EV experiment than in the tIR experiment. To ensure accurate comparison of spectra, the tIR spectrum is cubic spline interpolated to have finer time steps for the normalization procedure. The 2D EV ($\tau_1 = 0$) spectrum is fitted to the tIR spectrum with the following Supplementary Equation:

$$S_{norm}^{2DEV}(\tau_1 = 0, \tau_2, \omega_3) = \alpha\left(S^{2DEV}(\tau_1 = 0, \tau_2, \omega_3)\right) + \beta \tag{2}$$

where a constant spectral intensity scalar (α) and a constant offset (β) are applied to the averaged 2D EV spectrum ($S^{2DEV}(\tau_1 = 0, \tau_2, \omega_3)$) and optimized in a nonlinear least squares fitting routine to obtain the tIR-normalized 2D EV spectrum ($S_{norm}^{2DEV}(\tau_1 = 0, \tau_2, \omega_3)$). Initial values are set to α =1 and β =0 in the fitting routine; we find $\bar{\alpha}$ = 0.97 (±0.06) and $\bar{\beta}$ =0.00015 (±0.00048) (where the bar denotes the average over the optimized parameters for all τ_2 spectra). While the optimized parameters indicate that minimal adjustment to the original 2D EV spectrum is needed (i.e., on average, the intensities of the difference signals are scaled by less than 5% and offset by less than 2% of the signal magnitude for the ν_{COO} mode), this procedure effectively corrects for instrumental noise present in the 2D EV data as shown in Supplementary Figure 5. The optimized scalar and offset for each τ_2 spectrum are then applied to the raw 2D EV spectra over the complete range of τ_1 composing the 2D EV data set using Supplementary Equation (2). Then the tIR-normalized 2D EV data undergoes the usual FT processing over τ_1 described in Supplementary Note 1 to obtain the tIR-normalized 2D EV spectrum for further analysis.

3.2 Isolating Coherent Oscillatory Features in τ_2-dependent Data

The remaining analysis focuses on the excited state carboxylate symmetric stretching mode (ν_{COO}) at $\omega_3 \cong 1328$ cm^{-1} which is vibronically coupled to two distinct excited MLCT states (MLCT$_A$ and MLCT$_B$) as observed in the 2D EV spectra in manuscript Figure 2(a) and detailed by us previously.[1] Thus, the 2D spectral regions of interest are the (ω_1, ω_3) areas of the greatest 10-15% signal for these two features which are used throughout the remaining analysis (highlighted by red boxes in Supplementary Figure 6, the (ω_1, ω_3) ranges are given in Supplementary Table 2). We note that using slightly different bounds for the 2D EV regions of interest in the 10-15% signal range do not alter the conclusions from the analysis. We assume that measured population kinetics within the first 10 ps of the excited state triplet relaxation do not vary considerably between MLCT$_A$ and MLCT$_B$ because the fits to the tIR time traces described in Supplementary Note 2 only require a single exponential decay (τ_{short} in Supplementary Table 1) in this τ_2 period to fit the data well. To remove the population kinetics from the 2D EV regions of interest, we first average the (ω_1, ω_3) data points within the region of interest at each τ_2 delay to obtain what is effectively a tIR time trace for each excited MLCT state that is resolved by the 2D EV experiment. We then fit the population kinetics of the averaged 2D regions of interest using Supplementary Equation (1) while fixing the time constants (τ_{short} and τ_{long}) to the values obtained from the tIR measurements (given in Supplementary Table 1). The fitted population kinetics are then subtracted from the 2D data points in each region of interest to isolate coherent oscillatory dynamics that are modulating the 2D EV signal corresponding to the vibronically coupled ν_{COO} and either MLCT$_A$ or MLCT$_B$.

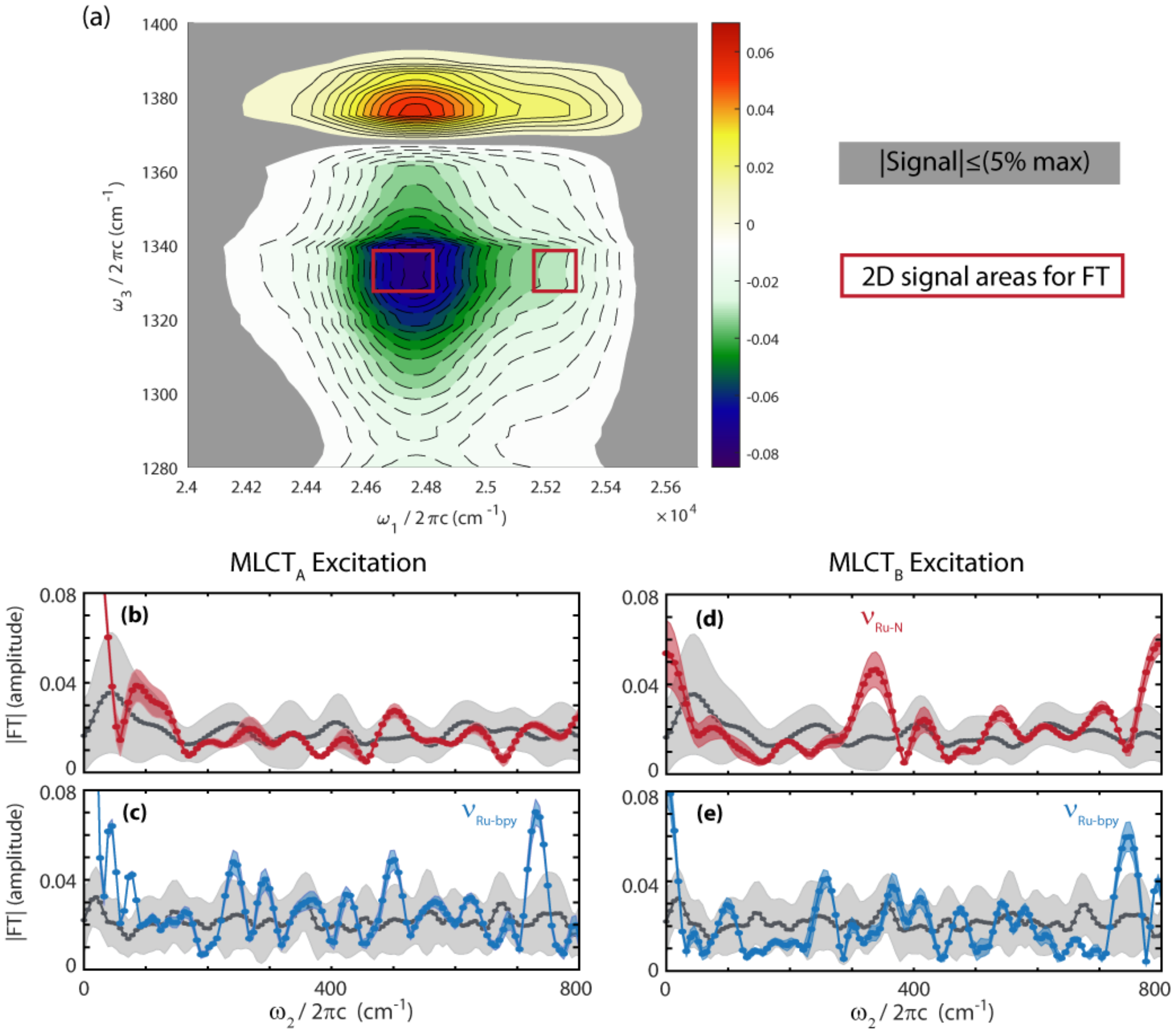

Supplementary Figure 6 Assessing Signal-to-Noise in ω_2 Spectra. (a) Averaged 2D EV spectrum over all τ_2 surfaces; gray shows background signal and red boxes highlight 2D EV regions of interest for vibronic couplings between ν_{COO} and both $MLCT_A$ and $MLCT_B$. Spectra from FT analysis on $0 \leq \tau_2 \leq 600$ fs data (b, red) and on $400 \leq \tau_2 \leq 1500$ fs data (c, blue) for $MLCT_A$ excitation. Spectra from FT analysis on $0 \leq \tau_2 \leq 600$ fs data (d, red) and on $400 \leq \tau_2 \leq 1500$ fs data (e, blue) for $MLCT_B$ excitation. Gray spectra in (b-e) are from FT analysis of the background signal shown in (a). The circles represent the average ω_2 spectrum and the shaded areas represent ± 1 standard deviation from the mean. Number of (ω_1, ω_3) points: $MLCT_A$ = 45; $MLCT_B$ = 24; Background (signal ≤ 5% max) = 3295.

Once population kinetics are removed, consequent FT analysis over the τ_2 delay of the 2D EV regions of interest provides a low-frequency spectrum, ω_2, of the τ_2-dependent 2D EV signal oscillations. As discussed in the manuscript, dynamics in the early time ($0 \leq \tau_2 \leq 600$ fs) and the later time ($400 \leq \tau_2 \leq 1500$ fs) periods involve different low-frequency modes. A double-sided tanh time domain filter is used with the form:

$$
F(\tau_2) = \begin{cases} \frac{1}{2} \cdot \tanh\left(\frac{\tau_2 - (\tau_c - \delta_{HW})}{B}\right) + \frac{1}{2}, & 0 \le \tau_2 \le \tau_c \\ -\frac{1}{2} \cdot \tanh\left(\frac{\tau_2 - (\tau_c + \delta_{HW})}{B}\right) + \frac{1}{2}, & \tau_c < \tau_2 \end{cases} \quad (3)
$$

to select the signals in either the earlier or later τ_2 periods. In Supplementary Equation (3), τ_c is the center of the tanh rise, δ_{HW} is the half-width of the double-sided tanh filter, and B is the rise time of the tanh function. The early time data is selected with τ_c = 270 fs, δ_{HW} = 350 fs, and B = 40 fs; the later time data is selected with τ_c = 910 fs, δ_{HW} = 550 fs, and B = 40 fs. The filtered data in each τ_2 period are zero-padded to 256 points prior to FT. The FT then resolves the ω_2 low-frequency spectrum and the absolute value spectra are analyzed (Supplementary Figure 6 b-e and manuscript Figure 2(c-d, f-g)).

The signal-to-noise of the ω_2 spectra is assessed by doing the same FT analysis on the respective τ_2 periods for 2D EV spectral regions that are effectively the background – i.e., where there is no 2D EV signal. Since there are regions of the 2D EV spectra with dynamic intensities during τ_2, we first averaged all of the 2D EV spectra at every τ_2 delay (resulting averaged 2D EV spectrum is shown in Supplementary Figure 6) and then we selected all (ω_1, ω_3) coordinates which have signal less than or equal to 5% of the 2D EV maximum signal in this averaged spectrum (0.0043). The solid gray contour shown in Supplementary Figure 6(a) highlights this background 2D EV region. The early time ($0 \leq \tau_2 \leq 600$ fs) ω_2 spectra are shown in Supplementary Figure 6(b, d) and the later time ($400 \leq \tau_2 \leq 1500$ fs) ω_2 spectra are shown in Supplementary Figure 6(c,e). The gray spectra show the average ω_2 spectrum and standard deviation of the background. The average ω_2 spectrum and standard deviation of the early time 2D EV signal for ν_{COO} coupled to both $MLCT_A$ and $MLCT_B$ are shown in red and the later time 2D EV signals are shown in blue. As discussed in the manuscript, these spectra provide clear evidence for the $\nu_{Ru\text{-}N}$ (ω_2 = 340 cm^{-1}) and the $\nu_{Ru\text{-}bpy}$ (ω_2 = 742 cm^{-1}) low frequency modes coupling with the vibronic eigenstates at different time periods within the first two picoseconds of the excited state triplet charge transfer and relaxation process. Other ω_2 regions in the spectra above the background noise approach the DC frequency (ω_2=0 cm^{-1}), which could be due to imperfect population kinetics subtraction, and as the Nyquist sampling limit is approached (ω_2=833 cm^{-1}). As a result, we do not consider the signals above $\omega_2 \cong$ 790 cm^{-1}.

Supplementary Table 2 *Regions of Interest for ω_2 Spectral Analysis*

	**ω_1 Range (cm^{-1}) [min:max]**	**ω_1 Resolution (cm^{-1})**	**# ω_1 "pixels"**	**ω_3 Range (cm^{-1}) [min:max]**	**ω_3 Resolution (cm^{-1})**	**# ω_3 "pixels"**
**$MLCT_A$**	[24600:24800]	14.1	15	[1330:1337]	2.6	3
**$MLCT_B$**	[25200:25300]	14.1	8	[1330:1337]	2.6	3

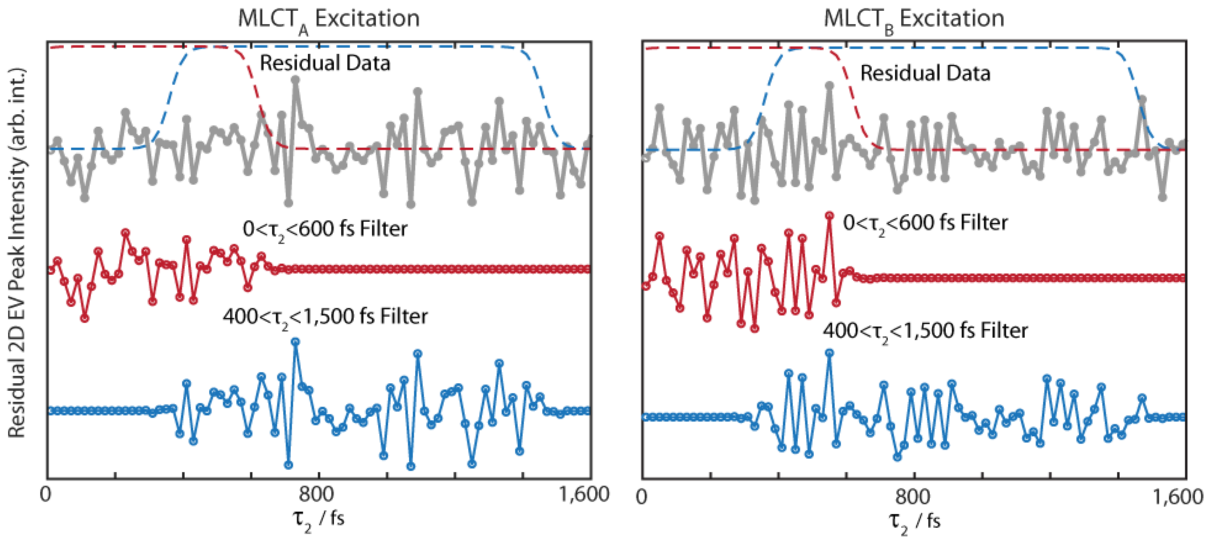

Supplementary Figure 7 Separating Early and Later Dynamics with Temporal Windowing. The early and later time τ_2-dependent residual intensities are shown offset in amplitude to more clearly see the raw data ($MLCT_A$: left, $MLCT_B$: right). The temporal windowing functions are shown in dashed lines on top of the residuals of the raw data; early time filtered data and temporal filter are shown in red, later time filtered data and temporal filter are shown in blue; the residuals of the raw data are shown in gray. The temporal windows are plotted in dashed lines on top of the raw data.

Supplementary Note 4. Characterizing the Initially Excited Vibrational Wavepacket

The initially excited state vibrational wavepacket composed of a coherence with the $\nu_{Ru\text{-}N}$ mode is characterized by extracting the spectral phase of the ω_2 spectrum, $\phi(\omega_2)$, fitting the phase to an nth order polynomial, and then obtaining the group delay, $\tau_d(\omega_2)$. Generally, a spectrum with arbitrary spectral phase variation can be written as $A(\omega-\omega_0)=\left|A(\omega-\omega_0)\right|e^{i\phi(\omega)}$. Here, the $\left|A(\omega-\omega_0)\right|$ factor is obtained by the absolute value of the Fourier transformed τ_2-dependent peak intensities over $0 \leq \tau_2 \leq 600$ fs (i.e., $\left|\mathrm{FT}(\tau_2)\right|$). The spectral phase and group delay are then obtained by [7]

$$\phi(\omega_2)=\tan^{-1}\left(\frac{\mathrm{Im}[\mathrm{FT}(\tau_2)]}{\mathrm{Re}[\mathrm{FT}(\tau_2)}\right) \tag{4}$$

$$\tau_d(\omega_2)=-\frac{d\phi(\omega_2)}{d\omega_2} \tag{5}$$

The group delay describes the time-dependence of the frequency components in a wavepacket, which in this case reflects the propagation of the wavepacket during τ_2 in the excited MLCT manifold during the early time period, $0 \leq \tau_2 \leq 600$ fs. We fit $\phi(\omega_2)$ over the spectral range of the ω_2 feature obtained in the FT analysis (ω_2 = 250-400 cm^{-1}) to polynomial functions of increasing order n, the coefficients ($P_{(n)}$) for each fit are given in Supplementary Table 3. A meaningful description of the wavepacket propagation is obtained by observing the consistencies in $\tau_d(\omega_2)$, obtained by numerical differentiation of the

polynomial function used in the fitting for different polynomial orders. The quartic polynomial (n=4) fit shown in Supplementary Figure 4e is used to obtain the group delay curve presented in manuscript Figure 4. The green curve in manuscript Figure 4 is identical to the red curve shown in Supplementary Figure 4f. As discussed in the manuscript, and shown in Supplementary Figure 8, all fits demonstrate consistent

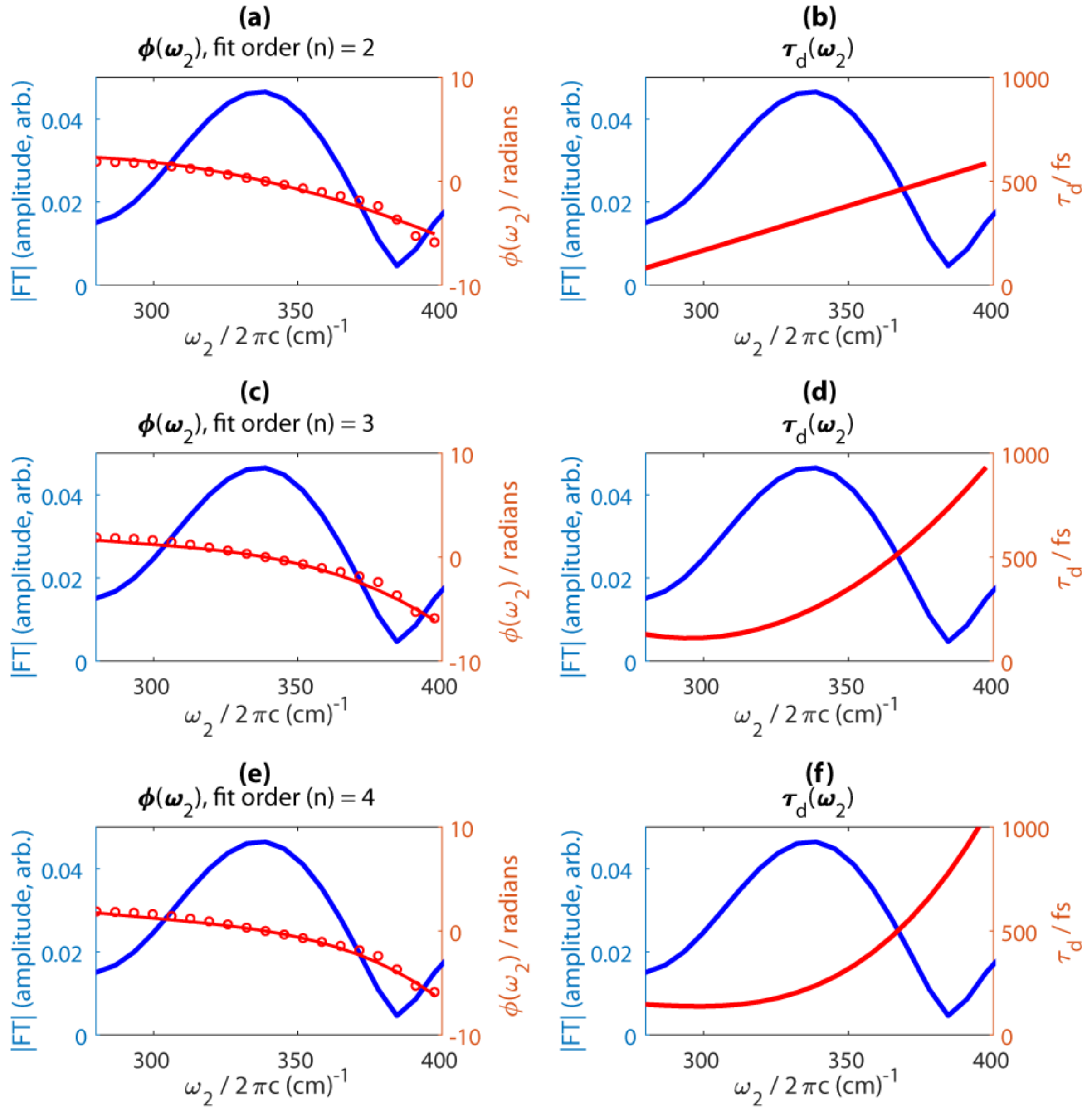

Supplementary Figure 8 Characterizing the Initially Excited Vibrational Wavepacket. The blue lines in all figures are the same ω_2 spectra for the $\nu_{Ru\text{-}N}$ wavepacket (ω_2 = 340 cm^{-1}) present during $0 \leq \tau_2 \leq 600$ fs. The figures in the left column (a,c,e) plot the spectral phase, $\phi(\omega_2)$, in red; experimental data is shown by the open circles and the solid lines are fits. The figures in the right column (b,d,f) plot the group delay, $\tau_d(\omega_2)$, in red, obtained by numerical differentiation of $\phi(\omega_2)$ as explained in Supplementary Equation 5. The different rows denote a different order of polynomial used to fit $\phi(\omega_2)$ during the analysis.

evidence for a blue shifting of the initially excited vibrational wavepacket during the first 600 fs of excited state relaxation. We place less emphasis on any interpretation based on the values of the coefficients for a particular fit given their dependence on the polynomial order, and instead consider the general trends observed across all fits. The same blue-shifting behavior is also observed through a short time Fourier transform (STFT) analysis of the earlier time data, as shown in Supplementary Figure 9 below. We give preference to the group delay analysis shown in the manuscript over the STFT because the blue-shifting is occurring during the course of only a few cycles of the $\nu_{Ru\text{-}N}$ vibration, which is also comparable to the ~ 600 fs lifetime of the wavepacket. Thus, an appropriate choice of windowing function becomes more difficult. Nevertheless, the consistency between the group delay analysis and the STFT confirms the blue-shifting of the $\nu_{Ru\text{-}N}$ wavepacket during early times.

Supplementary Table 3*: Spectral Phase ($\varphi(\omega_2)$) Fitting with n^{th}-order Polynomials*

Fit Order (n)	$P_{(0)}$ (rad)	$P_{(1)}$ (rad · s)	$P_{(2)}$ (rad · s^2)	$P_{(3)}$ (rad · s^3)	$P_{(4)}$ (rad · s^4)	Goodness of Fit (R^2)
2	-46.68	1.12 x10^3	-1.14 x10^4	-	-	0.9705
3	113.73	-7.00 x10^3	1.24 x10^4	-7.42 x10^5	-	0.9923
4	-153.04	1.10 x10^4	-3.29 x10^5	4.27 x10^6	-2.06 x10^7	0.9932

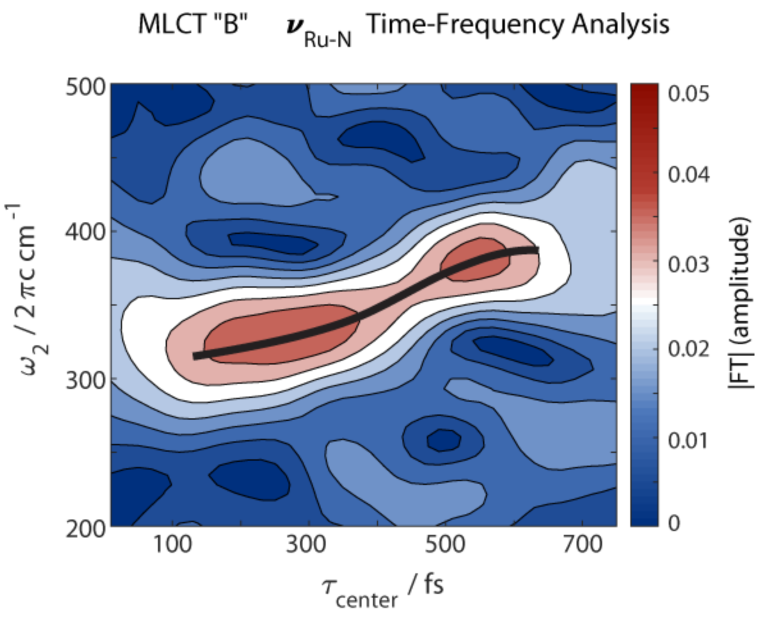

Supplementary Figure 9 Short Time Fourier Transform Analysis of Initial Wavepacket. Analysis performed using the same windowing function used in the later time STFT analysis (double-sided tanh) discussed in more detail in supplementary note 6, but with a window FWHM of 500 fs that has τ_{center} delays varied through the corresponding earlier delay times 0≤ τ_2 ≤ 600 fs. The thick solid black line is a guide to the eye to highlight the blue-shifting of the $\nu_{Ru\text{-}N}$ center frequency. Measured in this way, the ω_2 vibrational frequencies blue-shift by ~60 cm^{-1} over the interval 250 ≤ τ_{center} ≤ 570 fs, consistent with the blue-shift measured by the spectral phase / group delay analysis in Supplementary Figure 8.

Supplementary Note 5 Redfield Theory and Nonsecular Contributions to Relaxation Dynamics

Redfield theory is often useful for describing the time evolution of molecular systems.[8-10] The Redfield equation for the reduced density matrix, $\rho_{ab}(t)$ (where subscripts a and b denote system eigenstates), is expressed

$$\frac{\partial \rho_{ab}(t)}{\partial t} = -i\omega_{ab}\rho_{ab}(t) + \sum_{c,d} R_{ab,cd}\rho_{cd}(t) \tag{6}$$

where $\omega_{ab} = \frac{E_a - E_b}{\hbar}$ and $R_{ab,cd}$ is the Redfield tensor describing the relaxation dynamics between different system eigenstates. The first term on the right side of Supplementary Equation (6) describes the system's evolution in isolation and the second term involving the Redfield tensor describes the system's interaction with a dissipative environment. The Redfield tensor can be expressed as

$$R_{ab,cd} = \Gamma^{+}_{db,ca} + \Gamma^{-}_{db,ca} - \delta_{bd}\sum_{\kappa}\Gamma^{+}_{a\kappa,\kappa c} - \delta_{ac}\sum_{\kappa}\Gamma^{-}_{d\kappa,\kappa b} \tag{7}$$

where

$$\begin{aligned} \Gamma^{+}_{db,ca} &= \frac{1}{\hbar^2}\int_0^{\infty} dt \left\langle \left\langle d \middle| H_{SB}(t) \middle| b \right\rangle \left\langle c \middle| H_{SB} \middle| a \right\rangle \right\rangle_B e^{-i\omega_{ca}t}, \\ \Gamma^{-}_{db,ca} &= \frac{1}{\hbar^2}\int_0^{\infty} dt \left\langle \left\langle d \middle| H_{SB} \middle| b \right\rangle \left\langle c \middle| H_{SB}(t) \middle| a \right\rangle \right\rangle_B e^{-i\omega_{db}t}. \end{aligned} \tag{8}$$

and the κ index goes over system eigenstates. The system-bath Hamiltonian, H_{SB}, connects the system and the environment and is defined as $H_{SB}(t) = e^{(i/\hbar)H_B t} H_{SB} e^{-(i/\hbar)H_B t}$ where H_B is the bath Hamiltonian. In Supplementary Equation (8), the thermal average over the bath is denoted $\langle \ldots \rangle_B$. The Secular Approximation can be understood by recasting the Redfield equation within the interaction picture

$$\frac{\partial \rho^{I}_{ab}(t)}{\partial t} = \sum_{c,d} R_{ab,cd}\rho^{I}_{cd}(t)e^{i(\omega_{ab}-\omega_{cd})t} \tag{9}$$

and noticing that the integral averages to zero when both sides of Supplementary Equation (9) are integrated over a time interval $\Delta t >> (\omega_{ab} - \omega_{cd})^{-1}$ due to destructive interference. The Secular Approximation is made when only the terms satisfying $\omega_{ab} = \omega_{cd}$ are kept. However, nonsecular elements of $R_{ab,cd}$, those in which $\omega_{ab} \neq \omega_{cd}$, may become important when $\omega_{ab} - \omega_{cd}$ is very small which occurs in systems with strong system-bath coupling and/or when relaxation dynamics involve several eigenstates with molecular couplings represented by these nonsecular terms.

The dynamics of interest in this investigation occur during τ_2 where many vibronic eigenstates are involved with the ultrafast intersystem crossing and relaxation within the triplet manifold. The center frequency and bandwidth of the pump pulse (see Supplementary Table 4) in these experiments is sufficient to excite the following elements of the density matrix ρ_{ab} where $a,b \in \{1,2,3,4,5\}$ for the excited manifold

eigenstate numberings as shown in manuscript Figure 3. The coherences observed during τ_2 result from excitation of density matrix elements in which $a \neq b$. However, the density matrix elements that correspond to the ν_{COO} vibrations measured in ω_3 are ρ_{38} and ρ_{17}. The vibrational probe center frequency and bandwidth require that the system be in a population (ρ_{33} or ρ_{11}), rather than a coherence, in order for the ν_{COO} to be detected at ω_3 = 1328 cm^{-1} throughout the duration of the experiments. In other words, the probe bandwidth requires the transitions induced by the third light-matter interaction (+**k**$_3$) to be $\rho_{11} \xrightarrow{+\mathbf{k}_3} \rho_{17}$ or $\rho_{33} \xrightarrow{+\mathbf{k}_3} \rho_{38}$ because it is insufficient to both collapse the initially excited coherence and induce the first vibrational excitation of the ν_{COO}, (e.g., $\rho_{35} \xrightarrow{+\mathbf{k}_3} \rho_{38}$). Thus, a coherence-to-population transfer must occur during τ_2 to justify the observed signals. This is a nonsecular relaxation pathway, $R_{35,33}$, where $\omega_{35} - \omega_{33} = \nu_{Ru\text{-}N}$ (340 cm^{-1}) that drives the early time excited state vibrational wavepacket dynamics ($0 \leq \tau_2 \leq 600$ fs); a representative double-sided Feynman Diagram for this pathway is given in Supplementary Figure 10(a).

Whereas for the later time relaxation dynamics the excitation pulse center frequency and bandwidth is less important for determining the observed coherence during τ_2, the probe pulse center frequency and bandwidth still restrict the observable transitions in the same way as at early times. Thus, coherence-to-population transitions $\rho_{14} \xrightarrow{\tau_2} \rho_{11}$ and $\rho_{36} \xrightarrow{\tau_2} \rho_{33}$ during τ_2 are still required to observe the signals during $400 \leq \tau_2 \leq 1500$ fs, as shown in Supplementary Figure 10(b). We note that the coherence-to-coherence transitions responsible for the observed oscillatory electronic character fall within the secular approximation because the $\nu_{Ru\text{-}bpy}$ vibrational mode is part of the τ_2 coherence detected with both MLCT$_A$ and MLCT$_B$ character during the later relaxation time period which results in $\omega_{14} = \omega_{36}$.

Supplementary Table 4: *Pump and Probe Beam Center Frequencies and Bandwidths*

Beam	Center Frequency ω_0 / 2πc (cm^{-1})	Full Width 50% Max Intensity (cm^{-1})	Full Width 20% Max Intensity (cm^{-1})
Broadband UV Pump	24805	1090	1400
Mid-Infrared Probe	1361	198	245

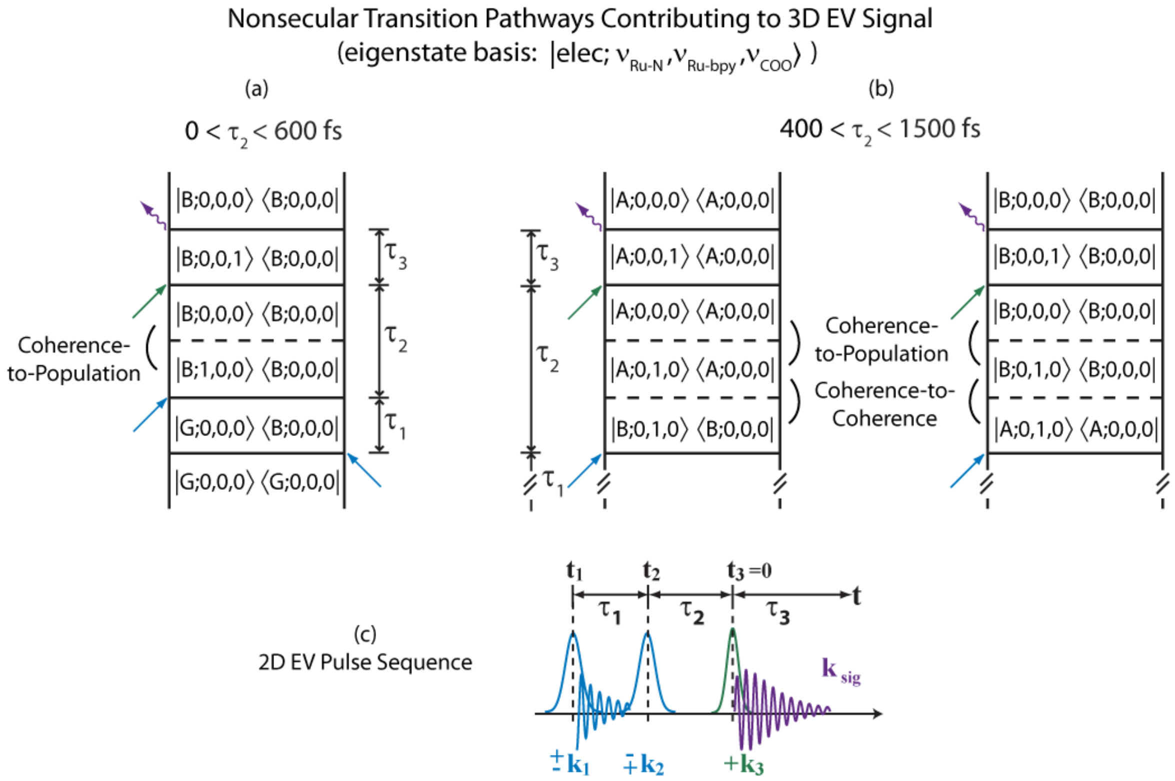

Supplementary Figure 10 Exemplary Nonsecular 3D EV Relaxation Pathways in $N3^{4-}$. (a) A representative Feynman Diagram of the excited state vibrational wavepacket present during early time relaxation dynamics which requires a coherence-to-population transition for detection. (b) Two representative Feynman Diagrams depicting the later time relaxation dynamics resulting in the oscillatory electronic character discussed in the manuscript, which still requires a coherence-to-population pathway for detection. The dashed lines connecting different states in the Feynman Diagrams represent transitions undergone without external perturbation from a laser pulse. (c) a 2D EV pulse sequence given for reference and interpretation of Feynman Diagrams.

Supplementary Note 6. Time-Frequency Methods Used in Later Time Data Analysis

The $\nu_{Ru\text{-}bpy}$ mode is the only prominently coupled low frequency mode apparent in our measurements during the later time relaxation dynamics, as shown in manuscript Figure 2(d,g), and it is clearly coupled to the ν_{COO} in both the $MLCT_A$ and $MLCT_B$. As Collini and co-workers have discussed,[11] the sliding window short time Fourier transform (STFT) is a useful time-frequency analytical method but it suffers when numerous frequency components with varying lifetimes contribute to a time-domain signal. This is due principally to an inherent uncertainty between temporal and frequency resolution in FT-based signal processing. The STFT uses a temporal window of a fixed width and a given temporal center to filter time-domain experimental data prior to Fourier transformation. In this way, a frequency spectrum for a temporally-localized portion of the time-domain data is obtained. This is repeated for many different temporal filter center positions to produce a two-dimensional time-frequency plot where the FT spectrum is plotted for every temporal filter center position. In the present analyses, ω_2=742 cm^{-1} is selected from the ω_2 dimension of the time-frequency plot for consideration as it corresponds to the vibrational mode

of interest, $\nu_{Ru\text{-}bpy}$, as identified by the ω_2 spectra shown in manuscript Fig. 2d and 2g obtained by considering all of the τ_2-dependent data in the later time period. As described below, we can reliably use a temporal filter to isolate the $\nu_{Ru\text{-}bpy}$ dynamics because this is the only sufficiently resolved mode in the ω_2 spectra for both $MLCT_A$ and $MLCT_B$ during later times. For time-domain signals containing multiple frequency components of interest that have significantly different periods of oscillation, the choice of a single temporal filter may favor a particular frequency component in the analysis over the others. Fortunately, that is not the case in our data.

In this analysis, only the $\nu_{Ru\text{-}bpy}$ is of primary interest as it is the strongest signal observed in ω_2 when all of the later time relaxation data is Fourier transformed (see manuscript Figure 2). Without a time-frequency analysis, the electronic oscillatory behavior of the $\nu_{Ru\text{-}bpy}$ coherence discussed in the main text would be missed. The ~45 fs period of the $\nu_{Ru\text{-}bpy}$ appears in 2-3 cycle bursts beginning in $MLCT_B$ and then oscillating between $MLCT_A$ and $MLCT_B$. Fortunately, this dynamic provides a straightforward choice of filtering window to be used in the analysis: a temporal filter with ~120 fs width sufficiently isolates each 2-3 cycle oscillation of the $\nu_{Ru\text{-}bpy}$ to clearly extract the oscillations in electronic population shown in the main text. The single coherence of interest with a well-defined duration in $MLCT_A$ and $MLCT_B$ render the STFT analysis a suitable method in our case. In Supplementary Figure 11 a comparison of time-frequency analyses using three different temporal windowing functions (double-sided hyperbolic tangent, Hanning, Gaussian) demonstrates that the choice of window function does not change the analysis or conclusions in any way. For all three functions, the full-width-at-half-max was set wide enough to span a single 2-3 cycle oscillation of $\nu_{Ru\text{-}bpy}$ when the window is centered over the coherence (examples shown for each MLCT state in Fig. SI 6.1). There is negligible difference in the FT time traces (see Supplementary Figures 11 (b,d,f)) among analyses using different windowing functions of consistent temporal width. Conversely, the temporal width of the filter can have a large impact on the obtained results using a STFT in the time-frequency analysis. Supplementary Figure 12 demonstrates the importance of an initial consideration about the nature of the time-domain signals that are of interest in a data set prior to conducting a time-frequency analysis. For a time-domain signal such as ours which contains transient and periodic coherences, a temporal filter that is too wide may result in a large offset in FT signal in addition to a phase relationship between oscillations that is shifted from the true phase relationship (e.g., see Supplementary Figure 12 (a,b)). As the filtering window shrinks, the FT signal passes through a range in which only the offset is observed because the oscillatory information is not isolated well enough by the filter (e.g., see Supplementary Figure 12 (c,d)). When the filter width approaches the duration of the transient coherences of interest, then the time-frequency analysis via the STFT produces a reliable result (e.g., see Supplementary Figure 12 (e-h)). The time-frequency analysis in this paper uses the double-sided hyperbolic tangent window with a 120 fs FWHM temporal width, as shown in Fig. 6.2 (g,h).

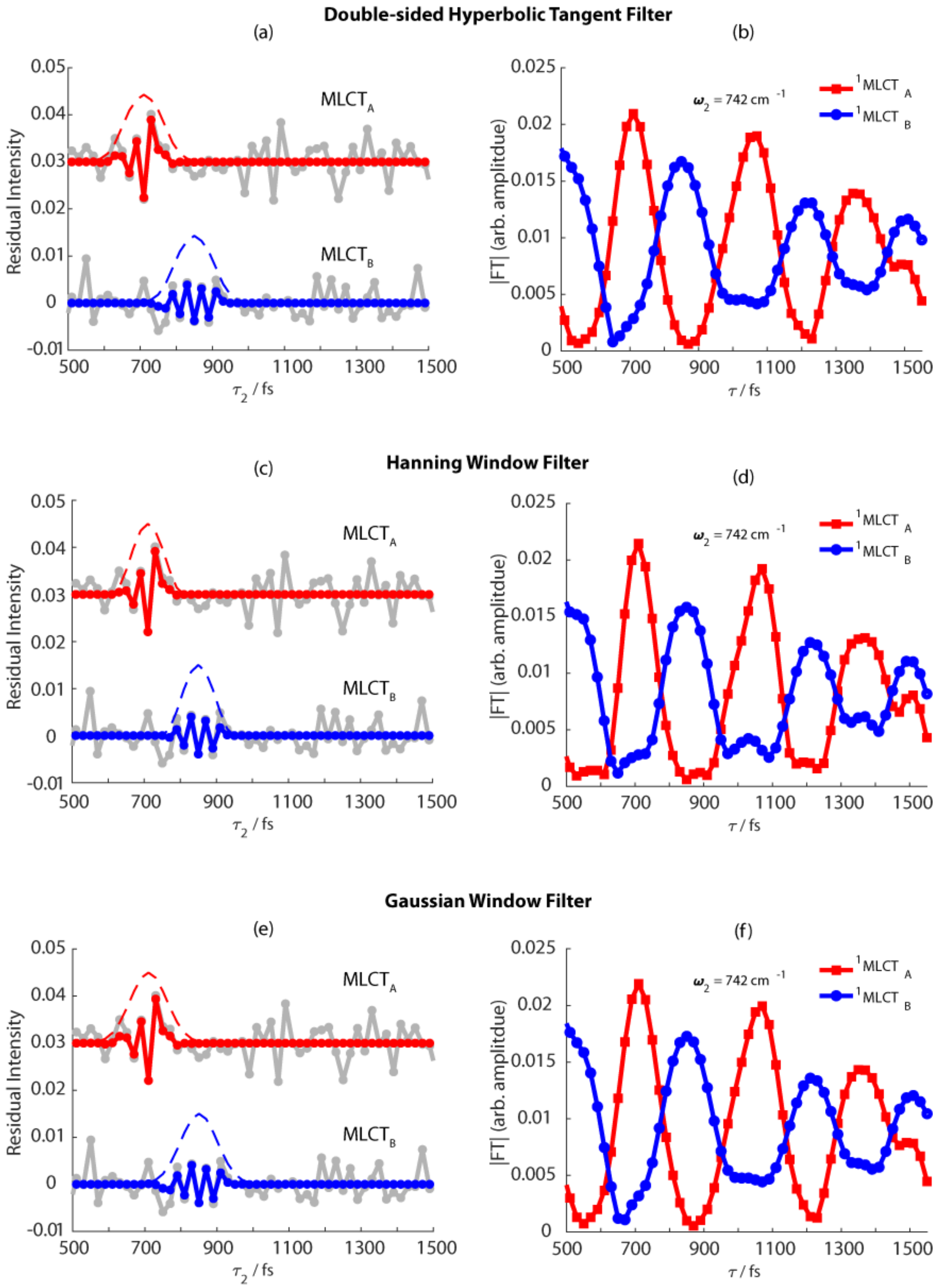

Supplementary Figure 11 Temporal Windowing Function Effects on Time-Frequency Analysis. The left column (a, c, e) show the 2D EV residual data (gray) for the signal regions of the ν_{COO} vibronically coupled with the MLCT$_A$ (red) and MLCT$_B$ (blue); the dashed lines show the temporal windowing functions used in the three time-frequency analyses for comparison. The right column (b, d, f) are the time-frequency analyses results from a short-time Fourier transform (details in the Supplementary Note 6 discussion) for MLCT$_A$ (red) and MLCT$_B$ (blue) at ω_3 = 742 cm^{-1}. A double-sided hyperbolic tangent window (a,b), Hanning window (c,d), and a Gaussian window (e,f) are compared.

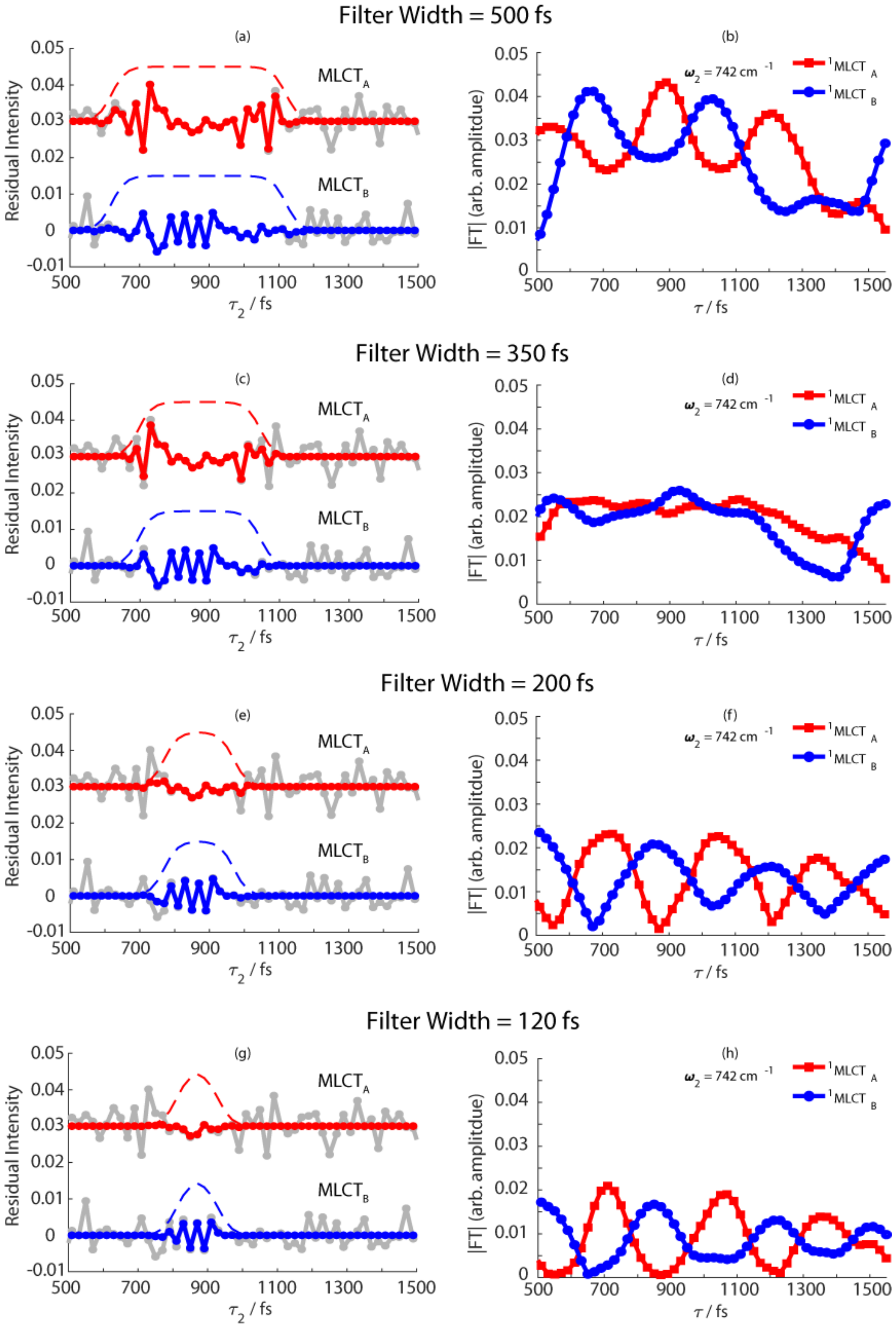

Supplementary Figure 12 Effect of Temporal Filter Width on Time-Frequency Analysis. The time-frequency analyses for varying filter widths (FWHM) are shown for the double-sided tanh window: (a,b) 500 fs, (c,d) 350 fs, (e,f) 200 fs, (g,h) 120 fs. The 2D EV residual time-domain data and temporal filtering is shown in the left column and the short time Fourier transformed data is plotted in the right column. The same coloration as Supplementary Figure 11 is used in this figure.

Supplementary Note 7. Nonadiabatic Internal Conversion Simulations: A Two-Level System

The time-dependent relaxation of the $\rho_{36}(\tau_2)$ and $\rho_{14}(\tau_2)$ coherences are simulated as shown in Fig 5c of the main text by solving the following system of differential equations:

$$\begin{pmatrix} \dot{\rho}_{36}(\tau_2) \\ \dot{\rho}_{14}(\tau_2) \end{pmatrix} = \begin{pmatrix} -i\omega_{36} - \Gamma_{36,36} & V_{NA}(\tau_2) \\ -V_{NA}(\tau_2) & -i\omega_{14} - \Gamma_{14,14} \end{pmatrix} \begin{pmatrix} \rho_{36}(\tau_2) \\ \rho_{14}(\tau_2) \end{pmatrix} \tag{10}$$

where $\omega_{36} = \omega_{14}$ = 742 cm^{-1} is the frequency of the $\nu_{Ru\text{-}bpy}$ coherence measured from the 3D EV experiments, $\Gamma_{36,36}$ = $\Gamma_{14,14}$ = 1/800 fs are the lifetimes of ρ_{36} and ρ_{14}, and V_{NA} = 50 cm^{-1} is the time-independent nonadiabatic coupling strength found to reproduce the observed 340 ± 40 fs periodicity of the oscillations between electronic character of the vibrational coherence amplitude between ρ_{36} and ρ_{14}. This observation is similar to the work of Dawlaty and co-workers[12] who found Rabi-like oscillations in the amplitudes of vibrational coherences in a hydrogen-bonded charge-transfer system, quinhydrone. The vibronic eigenstates that compose an effective Hamiltonian for the aqueous N3^{4-} studied here are defined in terms of both electronic and vibrational coupled degrees of freedom, providing a more complete view of an effective Hamiltonian capturing the dynamics of interest.

Supplementary Note 8. Negligible Frequency Shifting of Peaks in ω_1

The peak maxima of the carboxylate vibrations, both the ground state bleach and the excited state absorption (ν_{COO}) do not show any significant peak shifting after the pulse overlap and throughout the τ_2 range probed in the 3D EV experiments. This provides evidence that the excited MLCT states are strongly spin mixed as no electronic features arise to suggest that transitions into different spin states is resolved in the data.

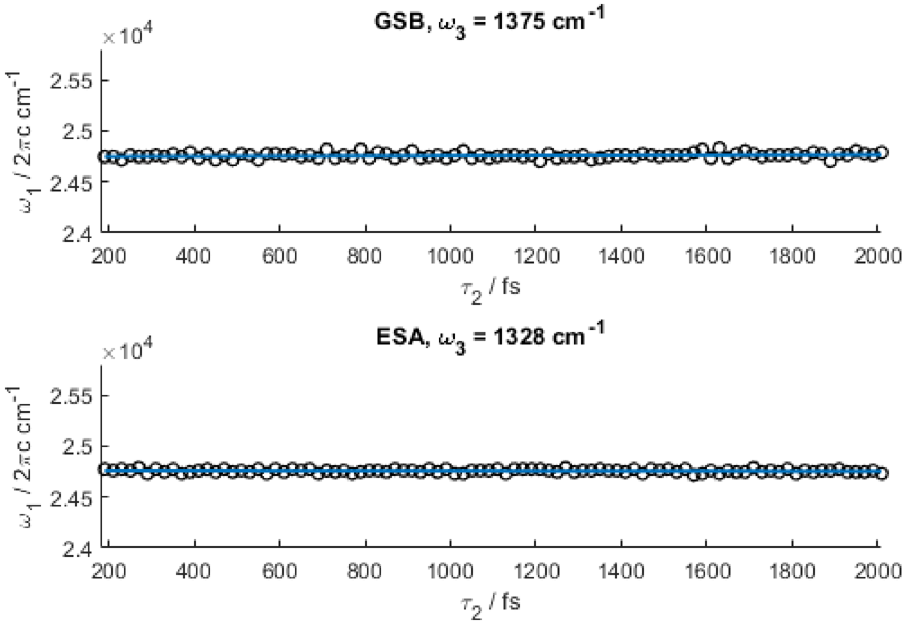

Supplementary Figure 13 Carboxylate ω_1 Peak Positions During τ_2. The ground state bleach (top) and excited state absorption (below, ν_{COO}) peak positions in ω_1 show negligible peak shifting over the range 190 < τ_2 < 2010 fs.

Supplementary Note 9. Calculated Low-Frequency Vibrational Modes

The calculated IR spectrum of the $N3^{4-}$ lowest energy triplet, T_0, is reproduced from *Gaynor et al.*[1] to display the full spectrum, including the low-frequency region (see zoom-in of the inset in Supplementary Figure 14a below). The normal mode vibrations with vector displacements provide examples of the several calculated modes in the $\nu_{Ru\text{-}N}$ (260-430 cm^{-1}) region and the $\nu_{Ru\text{-}bpy}$ (710-755 cm^{-1}) region with the respective vibrational character of the low-frequency vibrations discussed in the main manuscript. Computational details for these spectra can be found in *Gaynor et al.*[1] and its Supporting Information.

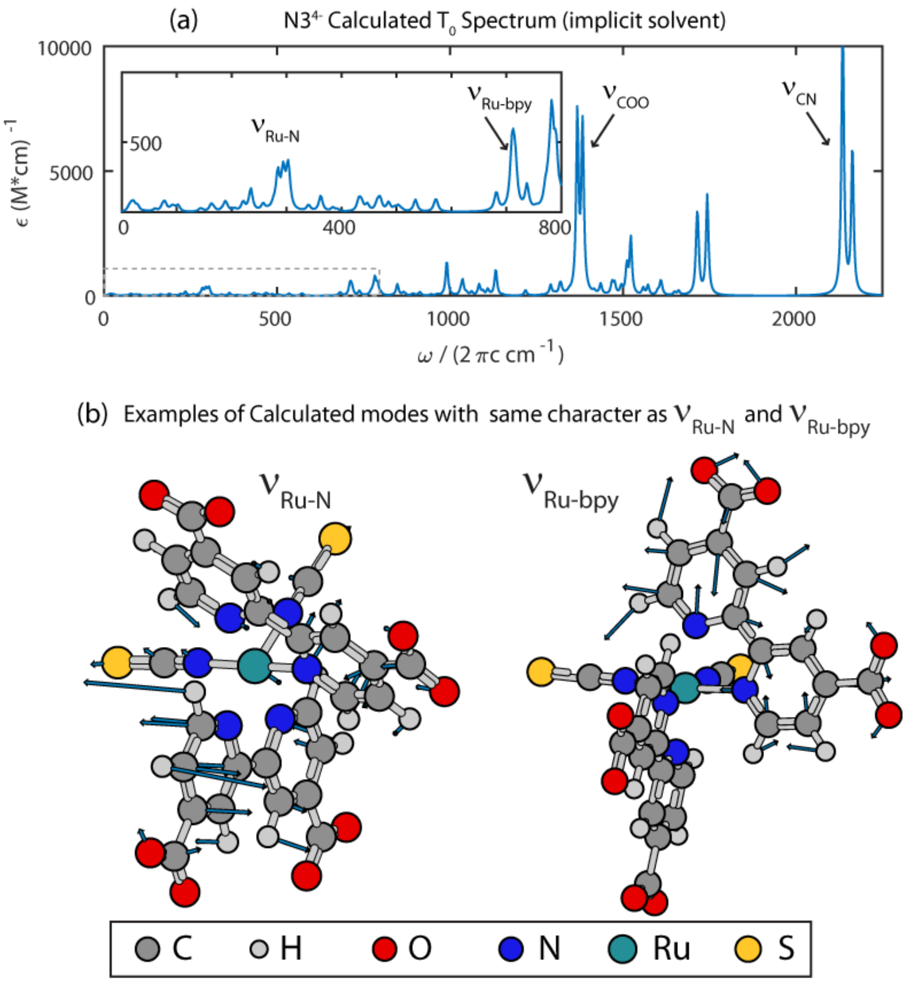

Supplementary Figure 14 (a) Calculated IR spectrum of the lowest energy triplet of $N3^{4-}$ with implicit solvent; inset shows a zoom-in of dashed gray spectral region. (b) Example normal modes from calculations displaying vibrational character of the $\nu_{Ru\text{-}N}$ and $\nu_{Ru\text{-}bpy}$ modes. Mode 39 (303 cm^{-1}) shown for $\nu_{Ru\text{-}N}$ and mode 67 (710 cm^{-1}) shown for $\nu_{Ru\text{-}bpy}$.

Supplementary References

1 Gaynor, J. D., Petrone, A., Li, X. & Khalil, M. Mapping Vibronic Couplings in a Solar Cell Dye with Polarization-Selective Two-Dimensional Electronic–Vibrational Spectroscopy. *J. Phys. Chem. Lett.* **9**, 6289-6295 (2018).

2 Gaynor, J. D., Courtney, T. L., Balasubramanian, M. & Khalil, M. Fourier transform two-dimensional electronic-vibrational spectroscopy using an octave-spanning mid-IR probe. *Opt. Lett.* **41**, 2895-2898 (2016).

3 He, P. *et al.* High-efficiency supercontinuum generation in solid thin plates at 0.1TW level. *Opt. Lett.* **42**, 474-477 (2017).

4 Lu, C.-H. *et al.* Generation of intense supercontinuum in condensed media. *Optica* **1**, 400-406 (2014).

5 Hamm, P. & Zanni, M. T. *Concepts and Methods of 2D Infrared Spectroscopy*. (Cambridge University Press, Cambridge, U.K., 2011).

6 Yetzbacher, M. K. *et al.* Spectral restoration for femtosecond spectral interferometry with attosecond accuracy. *J. Opt. Soc. Am. B* **27**, 1104-1117 (2010).

7 Weiner, A. M. *Ultrafast Optics*. (Wiley, Hoboken, New Jersey, 2009). (see pages 59-60 and Figure 3.15 on page 114 for discussions of spectral phase and group delay).

8 May, V. & Kuhn, O. *Charge and Energy Transfer Dynamics in Molecular Systems*. 2nd edn, (Wiley, Weinheim, 2004).

9 Egorova, D., Kuhl, A. & Domcke, W. Modeling of ultrafast electron-transfer dynamics: multi-level Redfield theory and validity of approximations. *Chem. Phys.* **268**, 105-120 (2001).

10 Redfield, A. G. in *Advances in Magnetic and Optical Resonance* Vol. 1 (ed John S. Waugh) 1-32 (Academic Press, 1965).

11 Volpato, A. & Collini, E. Time-frequency methods for coherent spectroscopy. *Opt. Express* **23**, 20040-20050 (2015).

12 Rury, A. S. & Dawlaty, J. M. Rabi-like vibrational coherence transfer in a hydrogen-bonded charge transfer material. *Phys. Rev. B* **95** (2017).
